# A Wideband Circularly Polarized Pixelated Dielectric Resonator Antenna

**DOI:** 10.3390/s16091349

**Published:** 2016-08-23

**Authors:** Son Trinh-Van, Youngoo Yang, Kang-Yoon Lee, Keum Cheol Hwang

**Affiliations:** School of Electronic and Electrical Engineering, Sungkyunkwan University, Suwon 440-746, Korea; jsonbkhn@gmail.com (S.T.-V.); yang09@skku.edu (Y.Y.); klee@skku.edu (K.-Y.L.)

**Keywords:** aperture-coupled feeding, circular polarization, pixelated dielectric resonator antenna, wide bandwidth

## Abstract

The design of a wideband circularly polarized pixelated dielectric resonator antenna using a real-coded genetic algorithm (GA) is presented for far-field wireless power transfer applications. The antenna consists of a dielectric resonator (DR) which is discretized into 8 × 8 grid DR bars. The real-coded GA is utilized to estimate the optimal heights of the 64 DR bars to realize circular polarization. The proposed antenna is excited by a narrow rectangular slot etched on the ground plane. A prototype of the proposed antenna is fabricated and tested. The measured −10 dB reflection and 3 dB axial ratio bandwidths are 32.32% (2.62–3.63 GHz) and 14.63% (2.85–3.30 GHz), respectively. A measured peak gain of 6.13 dBic is achieved at 3.2 GHz.

## 1. Introduction

Currently, with the rapid growth of portable electrical devices, wireless power transfer (WPT) is on the forefront of electronics technology [1,2,3]. The WPT system is intended to transmit power from one device to another through free space without the use of wires or cables. Generally, two main techniques are considered for WPT systems; near-field inductive coupling and far-field radio-frequency (RF) methods. With WPT using the near-field inductive coupling technique, power is transmitted by magnetic inductive coupling between transmitting and receiving coils or wires. However, this technique is only capable of transferring power over short distances. In contrast, with WPT based on the far-field RF technique (referred to as far-field WPT), power is transmitted by electromagnetic radiation, which allows the transfer of electrical energy over longer distances. This feature makes the far-field method an attractive WPT option [4].

The key element of any far-field WPT system is the antenna, which is used to capture the radiated electromagnetic waves. The performance of far-field WPT systems strongly depends on how well the antennas are designed, as well as the antenna characteristics, such as the gain, bandwidth, impedance matching, and polarization. Several studies have investigated far-field WPT methods with metallic antennas, specifically microstrip patches and dipole antennas [5,6]. However, the efficiency of metallic antennas is reduced significantly in the microwave frequency range or higher due to metallic losses. On the other hand, dielectric resonator antennas (DRAs), which are considered as non-metallic antennas, exhibit several advantages compared to metallic antennas. DRAs provide high radiation efficiency, a compact size, a relatively wide bandwidth, and ease of excitation [7]. Therefore, DRAs are a promising candidate for far-field WPT systems. Compared to linearly polarized DRAs, circularly polarized (CP) DRAs are preferred because they can mitigate the losses caused by propagation effects and misalignment between the transmitting and receiving antennas [8]. Therefore, CP DRAs have attracted much attention recently [9,10,11,12,13,14,15]. To generate circular polarization by DRAs, most studies focused on modifying the excitation schemes or on utilizing dielectric resonators (DRs) with special geometries. CP DRAs with various excitation techniques using a dual feed with a 90° phase difference [9], a parasitic patch [10], and a modified cross-slot [11] have also been introduced. Several studies have applied DRs with special geometries to realize circular polarization; these include a grooved rectangular DR [12], a trapezoidal DR [13], and a Spidron fractal DR [14].

In another work [15], a CP DRA was realized by stacking two rectangular DR layers with a rotating angle relative to the adjacent layers.

In this paper, we propose a wideband CP antenna using a pixelated DR which is discretized into 8 × 8 grid DR bars with different heights. A real-coded genetic algorithm (GA) of the type widely used to solve many antenna optimization problems [16,17] is then utilized to estimate the optimal heights of the 64 DR bars to produce circular polarization. An aperture-coupled feeding technique through a narrow rectangular slot is used to excite the proposed antenna. The paper is organized as follows. The design concept of the proposed antenna is introduced in Section 2. Section 3 then presents the experimental results as well as a comparison between measurement and simulation results. Finally, the conclusion is given in Section 4.

## 2. Antenna Design

**Figure 1:**
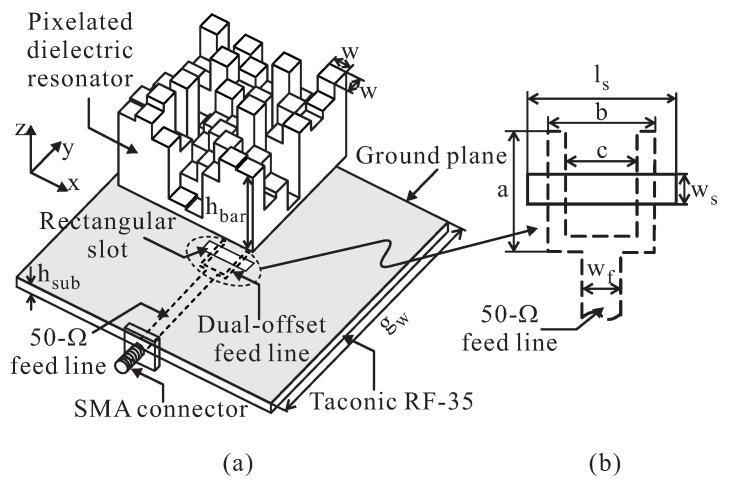
Geometry of the proposed antenna: (**a**) Exploded 3-D view; (**b**) Feeding configuration. SMA: SubMiniature version A.

Figure 1a shows the geometry of the proposed DR and feeding structure. The antenna consists of a pixelated DR, a feeding line, a Taconic RF-35 dielectric substrate (with a thickness $h_{sub}$ = 1.52 mm, a relative dielectric constant of $\epsilon_{r}$ = 3.5, and a loss tangent of tan *δ* = 0.0018), and a square ground plane. A rectangular slot with dimensions $w_{s}$ × $l_{s}$ = 3.6 mm × 21.4 mm is etched from the ground plane. The feeding line, consisting of a dual-offset feed line and a 50-Ω microstrip feed line, is mounted onto the bottom layer of the dielectric substrate (see Figure 1b). The dual-offset configuration is employed to obtain better impedance matching [15]. A pixelated DR with a width of 57.6 mm, a length of 57.6 mm, and a dielectric constant of 9.8 is discretized into 8 × 8 grid bars. Each bar has dimensions of *w* × *w* = 7.2 mm × 7.2 mm. The real-coded GA is used to optimize the heights of the 64 DR bars ($h_{bar}$) so as to realize wideband CP operation. In the implementation of the real-coded GA, a chromosome is encoded as a vector of 64 variables that are bound in a given range of 2 mm to 32 mm. Each variable represents the actual height of one DR bar; therefore, the GA yields the optimal heights of the DR bars after optimization. GA optimization is performed with 1000 iterations, a population of 20, a mutation rate of 0.15, and with the single-point crossover scheme. Table 1 summarizes the resultant optimal heights ($h_{bar}$) of the 64 DR bars, which generate right-handed circular polarization (RHCP). The other parameters are as follows (units: mm): $g_{w}$ = 140, *a* = 24.4, *b* = 16.8, *c* = 14, and $w_{f}$ = 3.3. The proposed antenna is analyzed and optimized using the ANSYS HFSS software. 

**Figure 2:**
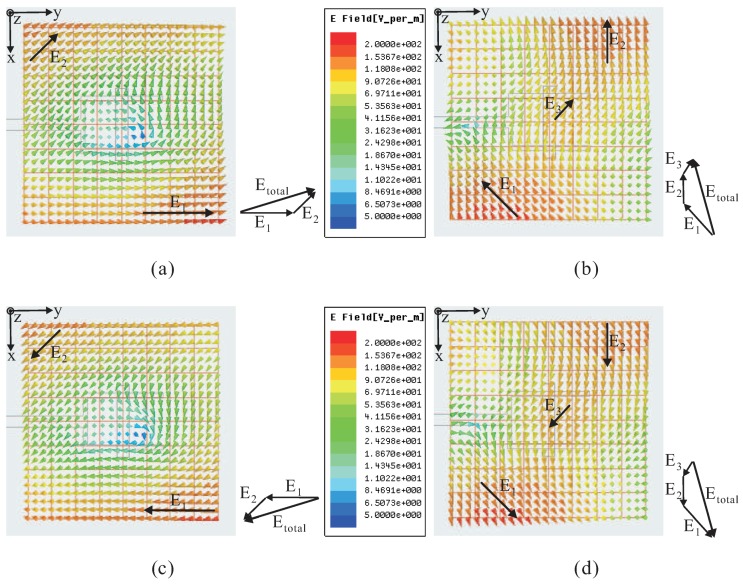
Simulated E-field distributions observed in the positive *z*-direction of the proposed dielectric resonator antenna (DRA) with time period *T* at 3.1 GHz: (**a**) *t* = 0; (**b**) *t* = *T*/4; (**c**) *t* = *2T*/4; (**d**) *t* = *3T*/4.

In order to verify the generation of RHCP, the E-field distributions in the positive *z*-direction of the proposed DRA are investigated at a frequency of 3.1 GHz. Figure 2 shows the simulated E-field distributions observed on the observation plane, located at a height of 48.4 mm (corresponding to one half of free space wavelength at 3.1 GHz) from the ground plane. Note that $E_{total}$ is the vector sum of the major E-field components. It is seen that at *t* = 0, the major E-field vectors generate a vector sum $E_{total}$ pointing from the lower left corner to the upper right corner. At *t* = *T*/4, the vector sum $E_{total}$ of the main E-field distributions points from the lower right corner to the upper left corner. This vector is orthogonal to that at *t* = 0 and rotates counterclockwise as the time *t* increases; thus, the RHCP is generated in the positive *z*-direction. 

**Figure 3:**
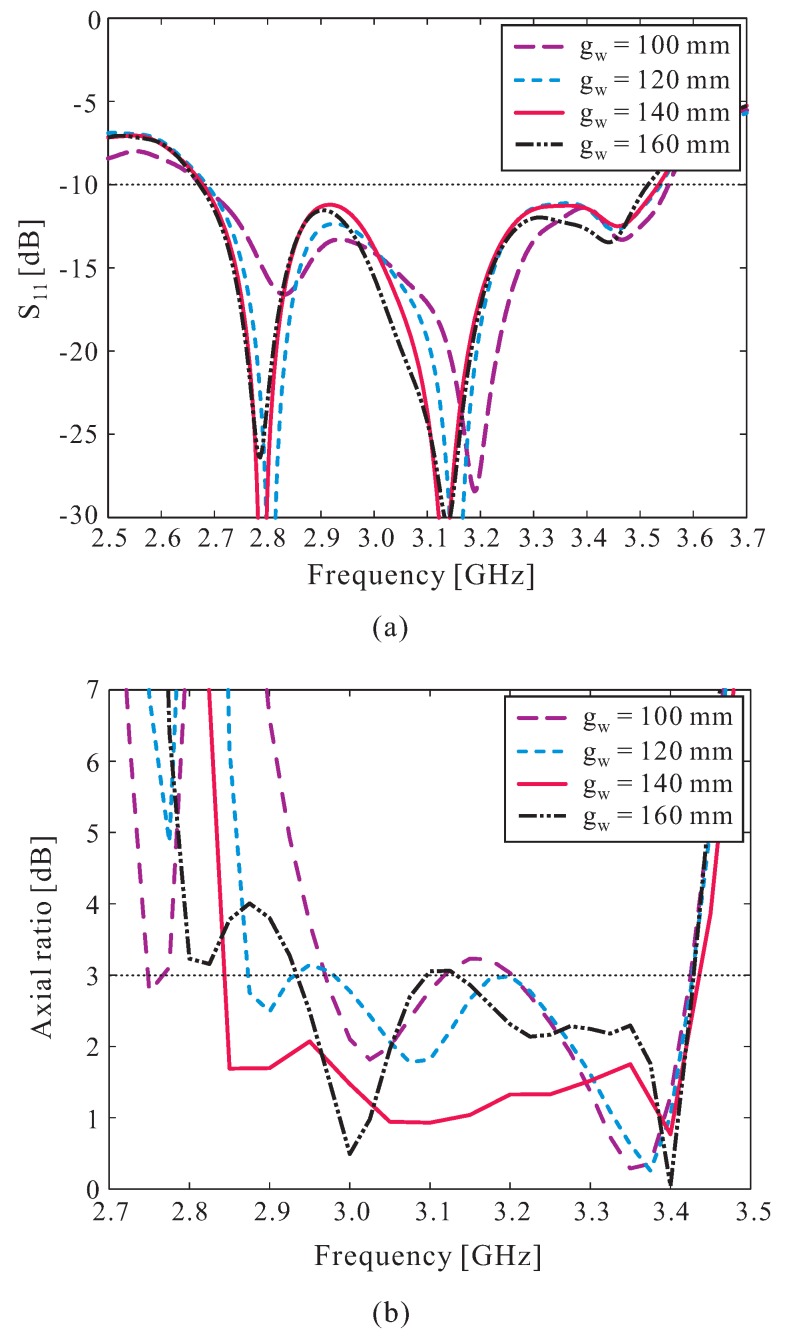
Effect of the ground plane size $g_{w}$ on: (**a**) reflection coefficient; (**b**) axial ratio (AR).

The effects of the ground plane size $g_{w}$ on the reflection coefficient and axial ratio (AR) performance are also investigated in a simulation, with the results illustrated in Figure 3. Note that the observed AR values indicate the positive *z*-direction (*θ* = 0°). Figure 3a shows that the ground plane size $g_{w}$ does not have a great effect on the overall reflection coefficient characteristic of the proposed antenna. Meanwhile, the variation of $g_{w}$ significantly affects the 3 dB AR bandwidth, as shown in Figure 3b. When $g_{w}$ increases, the levels of ARs at the middle frequency range are decreased. These levels essentially decrease to less than 2 dB when the value of $g_{w}$ equals 140 mm. In addition, the AR performance at the lower frequency is improved when the value of $g_{w}$ increases, forming a wider AR bandwidth. However, the level of AR within the lower frequency range increases again as the value of $g_{w}$ continues to increase. The value of $g_{w}$ is finally set to 140 mm, as that value provides the widest 3 dB AR bandwidth.

## 3. Experimental Results and Discussion

**Figure 4:**
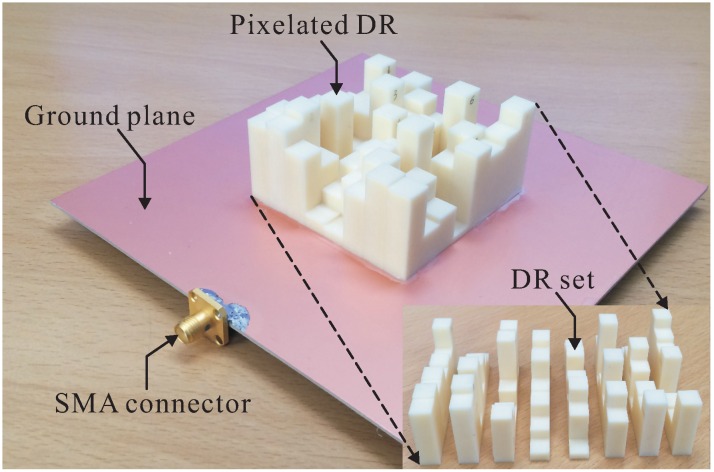
Photograph of the fabricated antenna.

**Figure 5:**
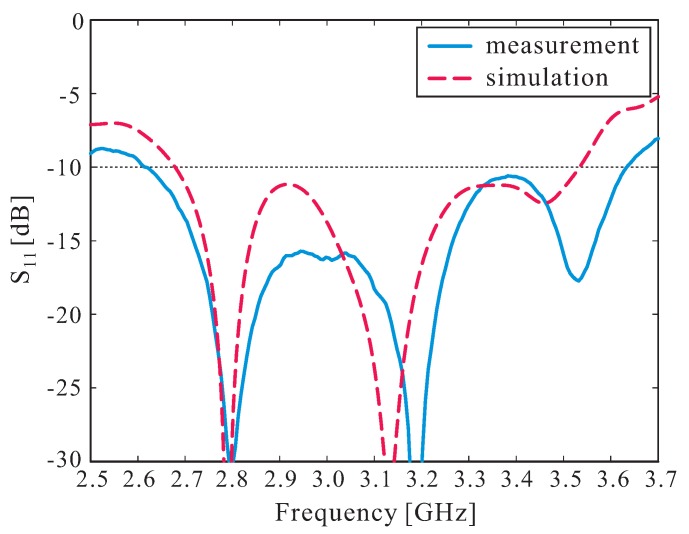
Measured and simulated reflection coefficients.

Based on the optimal parameters, the proposed antenna was fabricated and tested. To fabricate the proposed DR, 99.5% alumina ceramic with a dielectric constant of 9.8 and a loss tangent of 0.0001 was used. To simplify the milling process, the DR was divided into eight DR sets, with each DR set including eight DR bars along the y-axis. These fabricated DR sets were then glued together, after which their bottom sides were attached to the ground plane. A photograph of the fabricated antenna is shown in Figure 4. The reflection coefficient was measured using an Agilent 8510C network analyzer. The measured and simulated reflection coefficients are illustrated in Figure 5. It can be seen that the measured and simulated −10 dB reflection bandwidths are 32.32% (2.62–3.63 GHz) and 27.65% (2.68–3.54 GHz), respectively. Good agreement is thus observed between the measured and the simulated results.

**Figure 6:**
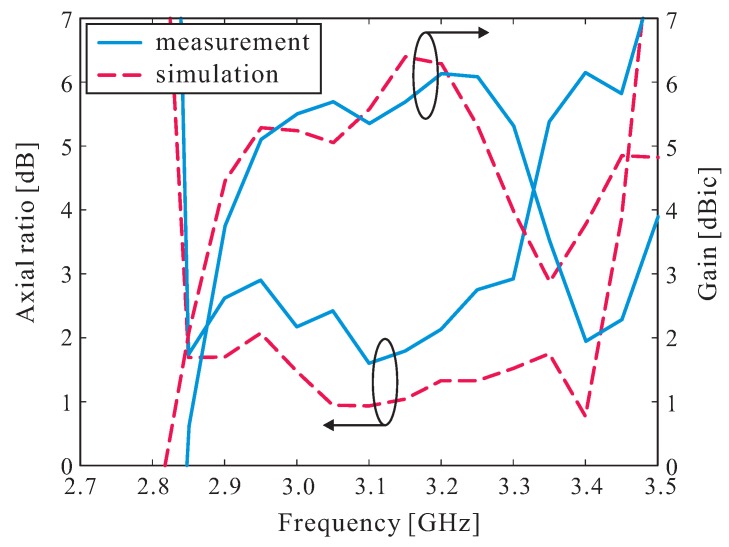
Measured and simulated axial ratios and right-handed circular polarization (RHCP) gains.

Figure 6 shows the measured and simulated axial ratios (ARs) and RHCP gains of the proposed antenna along the broadside direction (*θ* = 0°). The measured and simulated 3 dB AR bandwidths are 14.63% (2.85–3.30 GHz) and 18.47% (2.85–3.43 GHz), respectively. It was also noted that a measured peak RHCP gain of 6.13 dBic is achieved at 3.2 GHz. The discrepancy between the measurement and the simulation at higher frequencies (from 3.25–3.45 GHz) is mainly attributed to fabrication tolerance, especially in relation to the gluing process of the DR sets.

Table 2 shows a comparison of the proposed antenna and antennas presented in previous works [12,13,14], in which DRs with special geometries were utilized. Some of the earlier antennas [12,13] reportedly had wider impedances and AR bandwidths compared to the proposed antenna. However, one case [12] involved a combination of the two CP structures of a grooved rectangular DR and a Spidron fractal slot to obtain a wide CP bandwidth, though its peak gain was lower than that of the proposed antenna. Another antenna [13] utilized a trapezoidal DR with a tall height of 0.44$\lambda_{0}$ ($\lambda_{0}$ is the wavelength corresponding to the center frequency of the AR band), while the overall height of the proposed pixelated DR was only 0.33$\lambda_{0}$. Compared to the antenna using a Spidron fractal DR [14], the proposed antenna with the pixelated DR presents a wider AR bandwidth and a higher peak gain.

**Figure 7:**
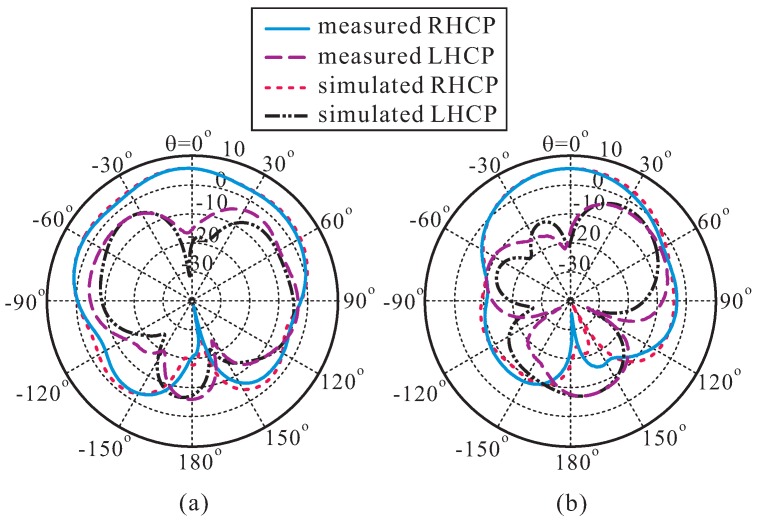
Measured and simulated radiation patterns at 3.1 GHz: (**a**) *xz*-plane; (**b**) *yz*-plane. LHCP: left-handed circular polarization.

Figure 7 depicts the measured and simulated radiation patterns of the proposed antenna on the *xz*- (*ϕ* = 0°) and *yz*- (*ϕ* = 90°) planes at 3.1 GHz. It was noted that left-handed circular polarization (LHCP) gain is 18.5 dB less than the RHCP gain in the broadside direction.

## 4. Conclusions

A microstrip-fed wideband CP antenna with a pixelated DR was proposed, fabricated, and tested. The DR was discretized into 8 × 8 grid DR bars with different heights, with the heights optimized by a real-coded GA to realize circular polarization. The experimental results proved that the proposed antenna exhibits a wide −10 dB reflection bandwidth of 32.32% (2.62–3.63 GHz), a 3 dB AR bandwidth of 14.63% (2.85–3.30 GHz), and a peak gain of 6.13 dBic. Compared to the LHCP gain, a high level of RHCP gain was also obtained in the broadside direction. Therefore, the proposed CP antenna, because it is capable of mitigating the polarization mismatch issue between the transmitter and receiver, is suitable for use as a wideband CP antenna element in far-field WPT applications.

## Figures and Tables

**Table 1:** The optimal heights ($h_{bar}$) of the 64 dielectric resonator (DR) bars in millimeters (Note that the cells in each column correspond to the positions of one DR bar along the *y*-axis).

The Optimized Values							
25.68	27.61	22.13	4.14	3.18	18.89	26.95	26.08
26.53	30.34	16.54	8.93	15.76	25.49	17.60	10.65
22.06	4.87	4.97	15.03	22.25	12.72	19.01	12.40
22.79	27.20	2.47	15.97	19.56	29.72	4.42	2.16
16.57	8.64	9.87	25.18	12.76	11.81	18.41	25.03
18.32	18.17	29.45	20.59	21.32	4.50	24.79	21.73
16.00	11.61	5.21	24.55	19.09	31.99	15.05	24.35
26.50	7.48	25.20	3.86	12.68	16.16	9.49	29.64

**Table 2:** Comparison of the proposed antenna with those in previous studies. Note that $\lambda_{0}$ represents the wavelength corresponding to the center frequency of the AR band.

Structure	Description	−10 dB Reflection Bandwidth (GHz)	3 dB AR Bandwidth (GHz)	Height ($\lambda_{0}$)	Peak Gain (dBic)
[12]	With a grooved rectangular DR	1.94–2.92 (40.33%)	2.30–2.92 (23.75%)	0.086	4.23
[13]	With a trapezoidal DR	2.88–4.04 (33.5%)	3.11–3.86 (21.5%)	0.44	8.39
[14]	With a Spidron fractal DR	4.32–6.30 (37.29%)	5.13–5.76 (11.57%)	0.13	3.16
Proposed antenna	With a pixelated DR	2.62–3.63 (32.32%)	2.85–3.30 (14.63%)	0.33	6.13

## References

[1] Liu X. (2015). A novel wireless power transfer-based weighed clustering cooperative spectrum sensing method for cognitive sensor networks. Sensors.

[2] Nguyen C.M., Kota P.K., Nguyen M.Q., Dubey S., Rao S., Mays J., Chiao J.-C. (2015). Wireless power transfer for autonomous wearable neurotransmitter sensors. Sensors.

[3] Liu L., Zhang R., Chua K.-C. (2013). Wireless information transfer with opportunistic energy harvesting. IEEE Trans. Wirel. Commun..

[4] Valenta C.R., Durgin G.D. (2014). Harvesting wireless power: Survey of energy-harvester conversion efficiency in far-field, wireless power transfer systems. IEEE Microw. Mag..

[5] Liu C., Guo Y.-X., Sun H., Xiao S. (2014). Design and safety considerations of an implantable rectenna for far-field wireless power transfer. IEEE Trans. Antennas Propag..

[6] Almoneef T.S., Sun H., Ramahi O.M. (2016). A 3-D folded dipole antenna array for far-field electromagnetic energy transfer. IEEE Antennas Wirel. Propag. Lett..

[7] Luk K.M., Leung K.W. (2003). Dielectric Resonator Antennas.

[8] Gao S., Luo Q., Zhu F. (2014). Circularly Polarized Antennas.

[9] Han R.-C., Zhong S.-S., Liu J. (2014). Broadband circularly polarised dielectric resonator antenna fed by wideband switched line coupler. Electron. Lett..

[10] Leung K.W., Ng H.K. (2003). Theory and experiment of circularly polarized dielectric resonator antenna with a parasitic patch. IEEE Trans. Antennas Propag..

[11] Zou M., Pan J. (2014). Wideband hybrid circularly polarised rectangular dielectric resonator antenna excited by modified cross-slot. Electron. Lett..

[12] Lee J.M., Kwon G., Song C.M., Lee K.-Y., Yang Y., Hwang K.C. (2015). Wideband circularly polarized Spidron fractal slot antenna with a grooved dielectric resonator. J. Electromagn. Waves Appl..

[13] Pan Y., Leung K.W. (2010). Wideband circularly polarized trapezoidal dielectric resonator antenna. IEEE Antennas Wirel. Propag. Lett..

[14] Altaf A., Yang Y., Lee K.-Y., Hwang K.C. (2015). Circularly polarized Spidron fractal dielectric resonator antenna. IEEE Antennas Wirel. Propag. Lett..

[15] Wang K.X., Wong H. (2015). A circularly polarized antenna by using rotated-stair dielectric resonator. IEEE Antennas Wirel. Propag. Lett..

[16] Ghatak R., Poddar D.R., Mishra R.K. (2009). Design of Sierpinski gasket fractal microstrip antenna using real coded genetic algorithm. IET Microw. Antennas Propag..

[17] Sato Y., Campelo F., Igarashi H. (2013). Meander line antenna design using an adaptive genetic algorithm. IEEE Trans. Magn..

